# D-2HG Inhibits Glutamine Synthetase Activity and Induces Senescence in IDH-Mutant Gliomas

**DOI:** 10.14336/AD.2025.0554

**Published:** 2026-08-04

**Authors:** Yuanlin Zhao, Jiankuan Shi, Yuan Yuan, Ying Yang, Xing Gao, Risheng Yang, Yuying Wang, Lijun Zhang, Jing Li, Peizhen Hu, Yingmei Wang, Chao Sun, Ruili Han, Yuqiao Xu, Zengshan Li, Zhe Wang, Yu Gu, Jing Ye

**Affiliations:** ^1^State Key Laboratory of Cancer Biology, Department of Pathology, Xijing Hospital and School of Basic Medicine, Fourth Military Medical University, Xi’an 710032, Shaanxi, China.; ^2^Xi'an International Medical Center Hospital Affiliated to Northwest University, Xi’an 710100, Shaanxi, China.; ^3^Southern Theater Command Air Force Hospital, Guangzhou 51030, Guangdong, China.; ^4^Department of Clinical Diagnosis, Tangdu Hospital, Fourth Military Medical University, Xi’an 710038, Shaanxi, China.; ^5^Department of Neurology, Tangdu Hospital, Fourth Military Medical University, Xi’an 710038, Shaanxi, China.; ^6^Department of Anesthesiology, Tangdu Hospital, Fourth Military Medical University, Xi'an 710038, Shaanxi, China

**Keywords:** IDH-mutant gliomas, D-2HG, glutamine synthetase, glutamine, senescence

## Abstract

IDH1/2 mutations are prevalent genetic alterations in gliomas that facilitate metabolic reprogramming and epigenetic modifications, which are essential for glioma progression. However, their exact contributions to tumorigenesis remain to be fully elucidated. Cellular senescence is a known precursor to tumorigenesis, and multiple oncogenes can initiate this senescence program. Our study demonstrated that the IDH1 mutation inhibits the proliferation of astrocytes and glioma cells, inducing cellular senescence through mechanisms involving DNA damage and increased production of reactive oxygen species (ROS). Notably, these effects were mitigated by the addition of exogenous glutamine. Within cells, glutamine synthetase (GS) serves as the sole enzyme responsible for glutamine synthesis. We found that D-2-hydroxyglutarate (D-2HG), an oncometabolite generated by mutant IDH enzymes, directly inhibits GS activity by binding to the glutamate site, substantially reducing endogenous glutamine production and exacerbating senescence in IDH1-mutant glioma cells. Additionally, in human glioma samples, a greater prevalence of GS-positive astrocytes was detected in IDH-mutant gliomas, likely providing adequate glutamine to sustain growth and mitigate senescence in these cells. Our findings suggest that D-2HG promotes senescence in IDH1-mutant glioma cells by inhibiting GS activity and that disrupting glutamine transport between astrocytes and glioma cells may constitute a promising therapeutic strategy for targeting IDH-mutant gliomas.

## INTRODUCTION

Isocitrate dehydrogenase 1 and 2 (IDH1/2) mutations are frequently observed in gliomas and serve as biomarkers with diagnostic, prognostic, and predictive significance [1]. In the early stages of tumorigenesis, IDH mutations lead to the production of the oncometabolite D-2-hydroxyglutarate (D-2HG), triggering a cascade of metabolic, epigenetic, and DNA damage-related alterations [2-9]. While IDH mutations are considered oncogenic drivers of tumor pathogenesis, neither IDH mutations nor D-2HG alone appear sufficient to induce tumorigenesis [10, 11]. Notably, IDH-mutant diffuse gliomas are associated with improved prognosis [12]. These findings underscore the need for further investigation into the precise role of IDH1 mutations in glioma progression.

Cellular senescence plays a dual role in cancer biology and treatment. On the one hand, certain oncogenes, such as H-Ras, PTEN, and Myc, contribute significantly to the induction of cellular senescence [13-17]. Conversely, senescence also serves as a protective mechanism against tumorigenesis, as chemotherapy and radiotherapy can induce senescence in cancer cells [18, 19]. Cancer cells are frequently subjected to diverse stressors that trigger senescence, including oncogenic signaling, replicative stress, hypoxia, reactive oxygen species (ROS), and nutrient deprivation. Glutamine (Gln) is vital not only for energy production but also as the primary nitrogen source for nucleic acids and as a precursor for glutathione (GSH) synthesis. Recent studies have demonstrated that increased glutaminolysis leads to ammonia production, which neutralizes acidic conditions and promotes the survival of senescent cells [20]. Glutamine synthetase (GS) is an exclusive enzyme in glutamine biosynthesis and is abandoned in astrocytes to support neurons through the glutamate–glutamine cycle [21, 22].

In IDH-mutant cells, glutamine-derived α-ketoglutarate (α-KG) serves as a key precursor for D-2HG production [23]. Previous studies have shown that IDH1 mutations significantly reduce glutamine and glutamate levels by inhibiting the transaminases BCAT1/2 [4]. In this study, we found that D-2HG competitively inhibits GS activity, reducing endogenous glutamine synthesis, which in turn exacerbates DNA damage and ROS accumulation, leading to cellular senescence. Notably, glioma-associated astrocytes (GAAs) were able to counteract senescence in IDH-mutant glioma cells by supplying adequate glutamine. This study reveals the link between glutamine biosynthesis and cellular senescence in IDH-mutant gliomas and provides a potential therapeutic strategy for their treatment.

## MATERIALS AND METHODS

### Animals and Glioma Tissue Collection

Mutant IDH1 conditional knock-in mice (gifts from Prof. Tak W. Mak) were crossed with GFAP-Cre mice (Cyagen Biosciences) to generate IDH1^lsl-R132H/+^; GFAP-Cre (IDH1-KI) mice, with IDH1^+/+^; GFAP-Cre (IDH1-WT) mice serving as controls. GS^flox/flox^ mice and Aldh1l1-CreERT mice were obtained from Cyagen Biosciences. All the mice were handled in accordance with the Guide for the Care and Use of Laboratory Animals, with experimental protocols approved by the Animal Ethics Committee of the Fourth Military Medical University (project number: IACUC-20180307). The animals were maintained on a 12-hr light/dark cycle with *ad libitum* access to water and standard chow. Animals with incomplete lesions were excluded from the study. Male mice weighing 20 g and 4 weeks of age were allowed. The animals were assigned to experimental groups using simple randomization.

Paraffin-embedded human glioma samples were collected from Xijing Hospital, the First Affiliated Hospital of the Fourth Military Medical University (China) (project number: KY20233192-1). IDH mutations were confirmed by Sanger sequencing and reviewed by pathologists from the Department of Pathology at Xijing Hospital.

### Astrocyte Isolation

Glial cells were isolated from neonatal mouse brains using a modified protocol based on established methods [24]. After the meninges were removed, the brain tissue was minced and digested with 0.25% trypsin for 15 minutes at 37°C. The resulting suspension was treated with 0.4 mg/mL DNase in EBSS to remove debris, filtered through a 300-mesh sieve, and cultured in DMEM containing 5.5 mM glucose and 10% FBS at 37°C. Upon reaching confluence, cultures enriched with astrocytes were shaken at 250 rpm for 24 hours to remove microglia and oligodendrocytes.

### Cell Lines and Growth Conditions

IDH1-wild-type U87 glioma cells (ATCC Lot: 63710285) were obtained from the American Type Culture Collection (ATCC), while U251 glioma cells from our laboratory were authenticated by STR profiling. The cells were cultured at 37°C in 5% CO_2_ in DMEM (Gibco, #12100046) supplemented with 10% fetal bovine serum (FBS) (Gibco, #10100147). For conditional experiments, DMEM (Gibco, #A1443001) containing 5.5 mM glucose and either low (0.2 mM) or high (4 mM) glutamine concentrations was used. The treatments included 5 mM L-methionine-DL-sulfoximine (MSO) (TargetMol, #T41 299), 1 μM 4-hydroxytamoxifen (Sigma-Aldrich, #H7904) and 5 μM V-9302 (Selleck Chemicals, #S8818).

### Inducible Expression of IDH1(R132H) in Glioma Cells

To induce the expression of mutant IDH1 in glioma cells, IDH1(R132H) was subcloned and inserted into pLVX-Tight-Neo vectors (Clontech) to create the pLVX-Tight-IDH1(R132H)-Neo lentiviral plasmid. The pLVX-TetOn-Puro plasmid and pLVX-Tight-IDH1(R132H)-Neo plasmid were co-transfected with psPAX2 and pVSVG into HEK-293T cells to produce lentiviruses. Infected U87 and U251 cells were then selected with puromycin and G418 for two weeks, and the expression of IDH1(R132H) was confirmed by immunoblotting with or without 10 µM doxycycline (Dox).

### Indirect Co-culture

Primary astrocytes were passaged in normal culture medium. Primary astrocytes were seeded in 60 mm dishes at a density of 0.5 × 10^6^ cells/cm². Conditional DMEM (Gibco, A1443001) containing 25 mM glucose, 0.2 mM glutamine, and 10% FBS was used to cultivate primary astrocytes. The medium was changed daily, and the culture medium was collected for the cultivation of U87 cells. Cell-conditioned medium (conditional DMEM with 25 mM glucose, 0.2 mM glutamine, and 10% FBS) was used as the control. U87 cells (IDH1-WT or IDH1-MU) were seeded in 60 mm dishes at a density of 0.5 × 10^6^ cells/cm² in normal medium. After the cells adhered, the indirect co-culture experiment was initiated by applying astrocyte-conditioned medium to the U87 cells. To prolong the co-cultivation period, 50% of the U87 culture medium was replaced daily. U87 cells were maintained in conditioned medium for one week prior to further assays, and 5 μM V-9302 (Selleck Chemicals, S8818) was added for one week.

### Cell Number Determination

Cells in 96-well plates were fixed with 100 μl/well of 4% paraformaldehyde for cell quantification. Fixed cells were stained with 100 μl/well of 0.5% crystal violet (Sigma) in absolute ethanol for 30 min. After removing the stain, the cells were rinsed three times in PBS and allowed to dry. Crystal violet was subsequently extracted with 100 μl of 10% acetic acid, and the absorbance was measured at 570 nm using a microplate spectrophotometer (Thermo Fisher Scientific 1510).

### Histology and Immunohistochemistry

Hematoxylin and eosin (H&E) staining, immunohistochemistry (IHC), and immunofluorescence (IF) were performed as previously described [25]. For IHC, the slides were incubated overnight at 4°C with primary antibodies, including anti-p53 (Abcam, #ab26), anti-p21 (ABclonal, #A22460), anti-p16 (ABclonal, #A11651), and anti-GFAP (Abcam, #ab7260), and the primary antibody-free group was used as the negative control. The tissue sections were then incubated with the relevant secondary antibody (Maixin Biotechnologies KIT-5002/KIT5005, China), followed by diamino-benzidine (DAB) visualization. Human glioma tissue was used as the positive control for P53 and GFAP staining, in accordance with the antibody datasheets. Human hepatocellular carcinoma tissue was used as the positive control for P21, and human lung squamous carcinoma tissue was used as the positive control for P16. Secondary antibody-only staining was performed as the negative control for all the immunohistochemical assays. For IF, the primary antibodies anti-GS (Abcam, #ab49873), anti-γ-H2A.X (CST, #9718), anti-53BP1 (Abcam, #ab175 933), and IDH1(R132H) (MXB Bio-technologies, #MAB-0733) were used. Secondary antibodies conjugated with Alexa Fluor 488 or 594 (Invitrogen, #181 4724 and #1820027) were applied, and the slides were mounted with DAPI-containing medium. Imaging was performed on an Olympus BX71 fluorescence microscope.

### SA-β-gal Staining

Senescence-associated β-galactosidase (SA-β-gal) staining was conducted using the Senescence β-Galactosidase Staining Kit (Beyotime, C0602) following the manufacturer’s instructions. For brain tissue staining, fresh brain tissue was sliced transversely into 3 mm sections, fixed in 4% paraformaldehyde (PFA) for 24 hrs, and then incubated in staining buffer at 37°C overnight. After washing with PBS, the stained brain slices were imaged. For cryosectioning, fresh brain tissue was embedded in Tissue-Tek O.C.T. (Sakura, 4583) and cryofrozen. Ten-micron-thick cryosections were fixed in 4% PFA for 30 minutes and incubated in staining buffer at 37°C overnight. For cell staining, the cells were fixed in 4% paraformaldehyde (PFA) overnight and then incubated in staining buffer at 37°C overnight. Images were acquired with an Olympus BX51 microscope.

### Immunoblot Analysis

Cells or tissues were lysed on ice in buffer containing 25 mM Tris-HCl (pH 7.6), 150 mM NaCl, 2 mM EDTA, 1% sodium deoxycholate, 0.5% SDS, 1% Triton X-100 and protease inhibitor cocktails. Proteins were separated by SDS–PAGE and transferred to PVDF membranes using standard protocols. The following primary antibodies were used: anti-p53 (HUABIO, # EM20603), anti-p53 (ABclonal, #A0263), anti-p53 (Abcam, #ab26), anti-p21 (ABclonal, #A22460), anti-p16 (ABclonal, #A11651), anti-GS (Abcam, #ab73593), anti-GLS (Invitrogen, #MA5-31538), anti-GLS2 (Invitrogen, #MA5-37732), anti-IDH1(R132H) (MXB Bio-technologies, #MAB-0733), anti-XPD (Abcam, #ab150362), and anti-POLD1 (Abcam, #ab86407), with β-actin (ABclonal, #AC026) and β-tubulin (ABclonal, #A12289) as the loading controls. The PVDF membrane was incubated with the corresponding horseradish peroxidase-conjugated secondary antibodies (Jackson #111-035-003 and #115-035-003) for 60 min at room temperature and developed with enhanced chemiluminescence (ECL) substrate.

### Glutamine Synthetase Activity

Glutamine synthetase (GS) was overexpressed in 293T cells through transfection with pCMV5-GS plasmids. Protein lysates were extracted from these GS-overexpressing cells, and GS activity in the lysates was measured in the presence of 10 mM D-2HG (Cayman Chemicals, #11605) using using a commercial GS assay kit (Solarbio, #BC0910) following the manufacturer's instructions. IDH1-mutant U87 and U251 glioma cells were cultured in standard DMEM supplemented with 10% FBS, and GS activity in these glioma cells was also assessed at 540 nm (Solarbio, #BC0910).

### Reactive Oxygen Species (ROS) Detection

U87 cells were cultured under various conditions, including treatment with 20 mM D-2HG, astrocyte-conditioned medium, 5 μM V-9302, and 5 mM MSO for 72 hours. The ROS levels were measured using a ROS Detection Assay Kit (Invitrogen, #1942272) following the manufacturer’s instructions. Briefly, the cells were incubated with 2’,7’-dichlorofluorescin diacetate (DCFH-DA) at a final concentration specified by the kit instructions for 30 minutes at 37 °C. After incubation, the cells were washed three times with serum-free culture medium and imaged using an Olympus IX-83 fluorescence microscope.

### NADPH Detection

Under the same conditions used for ROS detection, 5×10^6^ U87 cells were collected, and NADPH levels were measured using a commercially available kit (Applygen, #E2035) following the manufacturer’s instructions. Sample blank controls were included, and optical densities at 450 nm (OD450) were recorded using a microplate spectrophotometer (Thermo Fisher Scientific, 1510).

### BrdU DNA Synthesis Assay

DNA synthesis was evaluated by measuring the uptake of 5-bromo-2’-deoxyuridine-5’-monophosphate (BrdU). The cells were trypsinized, seeded onto sterile coverslips, and allowed to settle. After 48 hours of serum starvation, the cells were incubated in complete medium for 4 hours and then fixed and permeabilized with 0.1% Triton for 10 minutes. The cells were labeled with BrdU (10 μM; Sigma-Aldrich) for 1 hour, incubated with an anti-BrdU antibody in serum-free medium for 1 hour at 37°C, and stained with 4’,6-diamidino-2-phenylindole (DAPI) for nuclear visualization. The stained cells were imaged using a fluorescence microscope (Olympus IX-83).

### Molecular Docking

Molecular docking studies were conducted using AutoDock Vina [26]. The crystal structure of glutamine synthetase (GS) (PDB ID: 2QC8) was retrieved from the Protein Data Bank [27]. All water and solvent molecules were removed, and hydrogen atoms along with Gasteiger charges were added using AutoDock Tools [26]. The 3D structures of D-2-HG, L-Glu, and L-Gln were obtained from the ZINC database (http://zinc.docking.org/) and converted to pdbqt format through Applied Chemistry Software Openbabel [28]. A docking grid was defined to encompass the active protein site.

### TCGA Database

The expression data for GLS, GLS2, and GS in mutant IDH1/2 low-grade gliomas (LGGs) were retrieved from The Cancer Genome Atlas (TCGA, PanCancer Atlas, 2024) and analyzed using GraphPad software.

### Gene Set Enrichment Analysis (GSEA)

To elucidate the pathways linked to senescence in IDH-mutant gliomas, key canonical pathways (CPs), including reactome oxidative stress-induced senescence and reactome DNA damage telomere stress-induced senescence from the C2 category, were screened. Gene expression data were obtained from the TCGA cohort (TCGA, PanCancer Atlas, 2024) using the cBioPortal platform (https://www.cbioportal.org). The log2 ratio between wild-type and IDH1/2-mutant gliomas was calculated, and normalized gene expression data were then analyzed using gene set enrichment analysis (GSEA) with GSEA Java (version 4.3.2, Broad Institute).

### Target Identification Using Drug Affinity Responsive Target Stability (DARTS)

U87 cells were lysed with M-PER reagent (Thermo Fisher Scientific) supplemented with protease inhibitors, followed by the addition of TNF buffer (50 mM Tris-HCl, 50 mM NaCl, and 10 mM CaCl_2_, pH 8.0). The lysates were incubated with specified concentrations of D-2HG for 1 hr at room temperature. The samples were then divided: one portion was treated with 0.03% pronase (from a 10 mg/mL stock solution), while the other was left untreated. The digestion was halted by the addition of 5× protein loading buffer, and the samples were immediately heated to 95°C for 15 minutes. Proteins were separated via SDS–PAGE, transferred to PVDF membranes, and probed with an anti-GS antibody (Abcam, #ab73593).

### Intracranial Glioma Xenografts

5-6-week-old BALB/c nude mice were obtained from the Experimental Animal Center of the Fourth Military Medical University (FMMU). Following anesthesia, U87 glioma cells (either IDH1-WT or IDH1-MU) (5 × 10^5^ cells per mouse) were stereotactically inoculated 2 mm lateral to the right meninges and 3 mm subdurally within the brain. Tumor-bearing mice subsequently received daily intraperitoneal injections of V-9302 (30 mg/kg). Seven days after cell inoculation, mice were anesthetized with sodium pentobarbital and transcardially perfused with 4% paraformaldehyde. Brains were then harvested, and intracranial tumors were examined by hematoxylin and eosin (H&E) staining. Tumor areas were quantified using ImageJ software.

**Figure 1.**
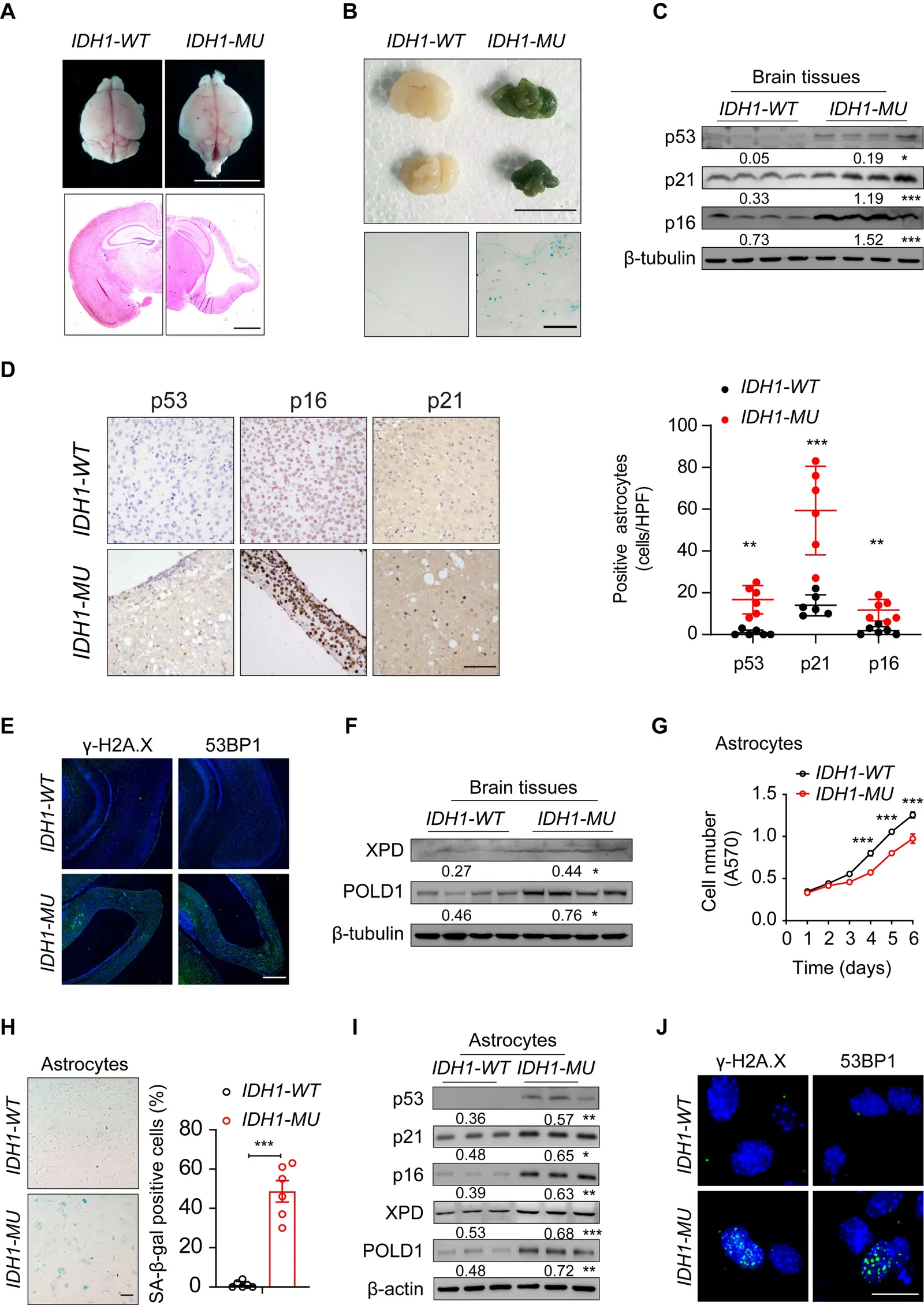
**IDH mutation accelerates astrocyte senescence in mice**. (**A**) Macroscopic images (upper) and H&E staining (bottom) of brains from 4-week-old wild-type (*IDH1-WT*) and astrocyte-specific IDH1(R132H) knock-in (*IDH1-MU*) mice. Scale bars are 10 mm (upper) and 1 mm (bottom) respectively, n = 6 mice per group. (**B**) SA-β-gal staining of brains from *IDH1-WT* and *IDH1-MU* mice. Scale bars are 8 mm (upper) and 100 μm (bottom) respectively, n = 6 mice per group. (**C**) Immunoblotting of p53, p21 and p16 in *IDH1-WT* and *IDH1-MU* brain tissues. (**D**) Immunohistochemical (IHC) staining and statistical analysis of p53, p21 and p16 in *IDH1-WT* and *IDH1-MU* brain sections respectively. Scale bar, 50 μm, n = 6 mice per group. (**E**) Immunofluorescence staining of γ-H2A.X (left) and 53BP1 (right) in *IDH1-WT* and *IDH1-MU* mouse brain tissues, and the nuclei were counterstained with DAPI (blue). Scale bar, 200 μm, n = 3 per group. (**F**) Representative immunoblotting and statistical analysis of XPD and POLD1 expression in *IDH1-WT* and *IDH1-MU* mouse brains, β-tubulin was used for normalization. (**G**) Growth curves of astrocytes from newborn *IDH1-WT* and *IDH1-MU* mouse brains, n = 4 per group. (**H**) SA-β-gal staining and statistical analysis of *IDH1-WT* and *IDH1-MU* astrocytes. Scale bar, 100 μm, n = 6 per group. (**I**) Immunoblotting and statistical analysis of p53, p21, p16, XPD and POLD1 in *IDH1-WT* and *IDH1-MU* astrocytes. n = 3 per group. (**J**) Immunofluorescence staining of γ-H2A.X and 53BP1 in primary *IDH1-WT* and *IDH1-MU* astrocytes, n = 3 per group. Scale bar, 20 μm. The percentage of SA-β-gal-positive cells relative to the total number of cells is presented (H). β-tubulin or β-actin was used for immunoblot normalization. All the data are presented as means ± standard errors of the mean (SEMs). Statistical significance was determined using unpaired Student’s *t* tests (D, F, H, I), Mann–Whitney tests (C, D, F) and one-way ANOVA (G) (*p < 0.05, **p < 0.01, ***p < 0.001).

### Statistical Analysis

All the results are expressed as the means ± standard errors of the means (SEMs) from at least three independent experiments. Statistical analyses were performed using GraphPad Prism (version 6.0, GraphPad Software, La Jolla, CA, USA). Data normality was assessed using the Shapiro–Wilk test. Comparisons were conducted using a two-tailed unpaired Student's *t* test (normal distribution statistics), the Mann–Whitney U test (nonnormal distribution statistics), one-way ANOVA (normal distribution statistics) or the Kruskal–Wallis test (nonnormal distribution statistics). For correlation analysis, data normality was evaluated with the D’Agostino–Pearson test, and the Pearson correlation coefficient was calculated.

## RESULTS

### Mutant IDH1 Knock-in Exacerbates Astrocyte Senescence in Mice

IDH1/2 mutations are frequently observed in lower-grade gliomas. Astrocyte-specific mutant IDH1(R132H) knock-in mice (IDH1^LSL-IDH1(R132H)/+^; GFAP-Cre, hereafter referred to as IDH1-MU) were generated to investigate their role in tumorigenesis. Our findings indicated that IDH1 mutation in astrocytes resulted in significant cortical structural damage, with marked cortical atrophy evident in 4-week-old *IDH1-MU* mice (Fig. 1A). We also observed a substantial increase in SA-β-gal-positive cells in the cortices of the *IDH1-MU* mice, suggesting that cell senescence was promoted (Fig. 1B). Furthermore, immunohistochemistry (IHC) and immunoblot analyses revealed increased expression of senescence markers, including p53, p16, and p21, in the IDH1-MU mice (Fig. 1C and D). Markers of DNA damage and repair, including tumor protein p53 binding protein 1 (53BP1), Phospho-Histone H2A.X (Ser139) (γ-H2A.X), ERCC excision repair 2 (XPD), and DNA polymerase delta 1 (POLD1) were also significantly upregulated in the brains of the *IDH1-MU* mice (Fig. 1E and F).

Primary astrocytes were subsequently isolated from newborn mice, and the results indicated that IDH1 mutation inhibited astrocyte growth (Fig. 1G). Staining revealed that the percentage of SA-β-gal-positive cells increased in IDH1-mutant astrocytes (Fig. 1H), and immunoblotting revealed that the levels of p53, p16, and p21 were elevated in these cells (Fig. 1I). Both immunoblot and staining analyses demonstrated increased levels of 53BP1, γ-H2A.X, XPD, and POLD1, suggesting the activation of DNA damage sensing and repair pathways in IDH1-mutant astrocytes (Fig. 1I and J). Collectively, these findings indicate that IDH1 mutation accelerates astrocyte senescence by inducing DNA damage.

### Mutant IDH1 Induces Senescence in Glioma Cells

**Figure 2.**
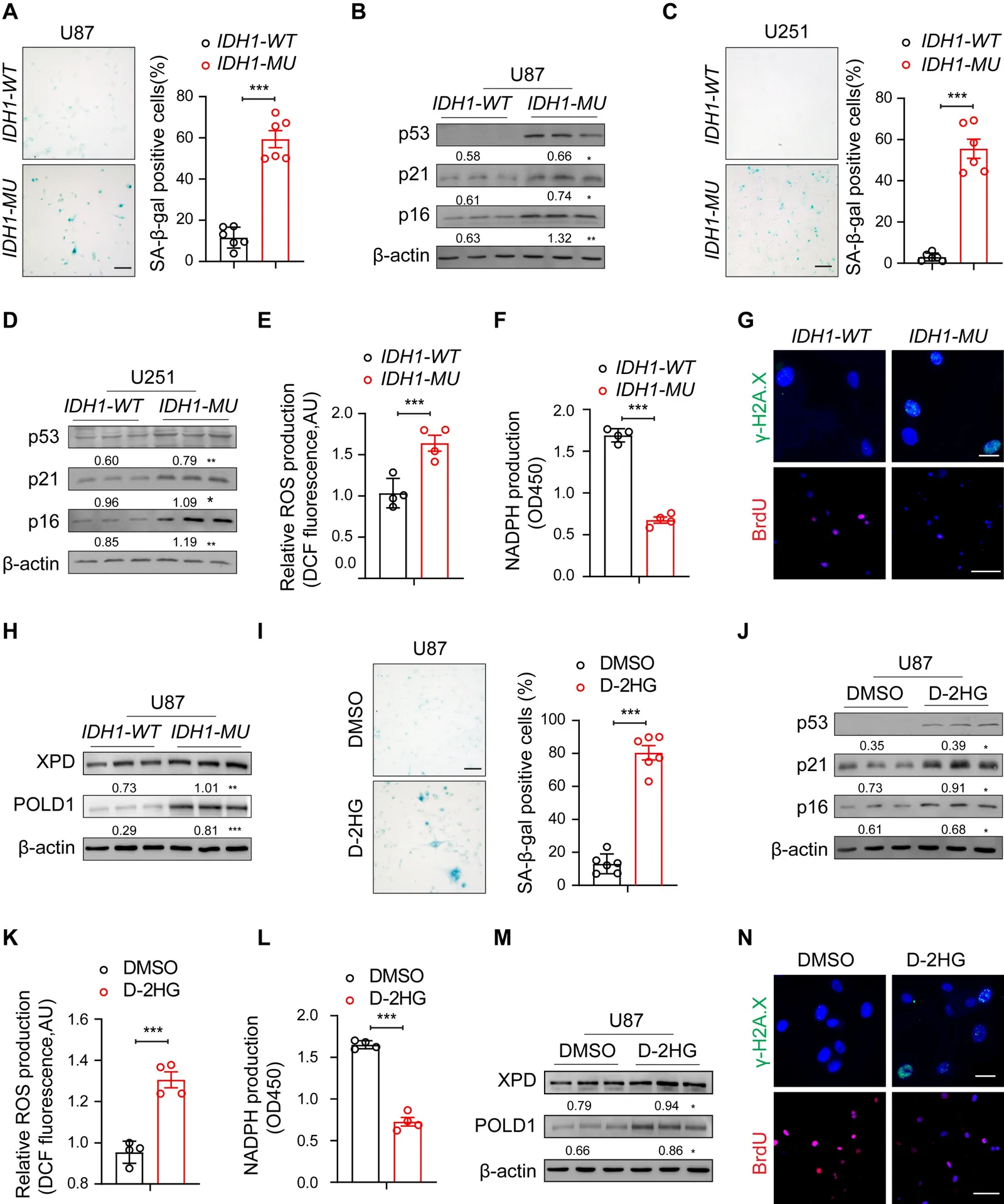
**IDH1 mutation induces senescence in the glioma cells**. (A&C) SA-β-gal staining of U87 and U251 glioma cells with wild-type (*IDH1-WT*) and mutant-IDH1 (*IDH1-MU*), respectively. Scale bar, 100 μm; n = 6 per group. (B&D) Immunoblotting and statistical analysis of p53, p21 and p16 in *IDH1-WT* and *IDH1-MU* U87 (B) and U251 (D) cells. (**E**) Relative ROS generation of *IDH1-MU* U87 cells compared with that in *IDH1-WT* U87 cells. The data are representative of three individual experiments with n = 4 per group (normalization: *IDH1-WT*). (**F**) NDAPH levels were analyzed in 5×10^6^ *IDH1-WT* and *IDH1-MU* U87 cells. The data are representative of three individual experiments with n = 4 per group. (**G**) Immunofluorescence staining of γ-H2A.X (upper) and BrdU (5'-bromo-2'-deoxyuridine ) (bottom) in *IDH1-WT* and *IDH1-MU* U87 cells, and the nuclei were counterstained with DAPI (blue). Scale bars are 20 μm (upper) and 100 μm (bottom). Data are representative of three individual experiments with n = 3 per group. (**H**) Immunoblotting and statistical analysis of the XPD and POLD1 expression in *IDH1-WT* and *IDH1-MU* U87 cells. (I&J) SA-β-gal staining (I) and immunoblotting of p53, p21 and p16 (J) in U87 glioma cells after treatment with 20 mM D-2HG for 72 hr. Data are representative of three individual experiments with n = 6 per group in I, and n = 3 per group in J. (**K-N**) Relative ROS generation (K), NADPH production (L), representative immunoblotting for XPD and POLD1 (M) immunofluorescence staining of γ-H2A.X (N, upper) and BrdU (N, bottom) of D-2HG-treated U87 glioma cells. Scale bars are 20 μm (upper) and 100 μm (bottom). The data are representative of three individual experiments with n = 4 per group in K and L and n = 3 per group in M and N. Data are presented as a percentage of SA-β-gal-positive cells to the total cell number in each group (A, C, I). β-actin was used for immunoblot normalization. All the data are presented as mean ± SEMs. Statistical significance was determined using unpaired Student’s t test (A, B, C, D, E, F, H, I, J, K, L, M) (*p < 0.05, **p < 0.01, ***p < 0.001).

Mutant IDH1 was overexpressed in U87 and U251 glioma cell lines to examine whether IDH mutations induce senescence in glioma cells. SA-β-gal staining demonstrated that IDH mutations promoted cellular senescence in both U87 and U251 cells (Fig. 2A and C), accompanied by increased expression of senescence markers, including p53, p21, and p16 (Fig. 2B and D). DNA damage and ROS production are the major contributors to cellular senescence [29-31]. Here, our findings revealed that IDH mutation elevated ROS levels and decreased NADPH production in glioma cells (Fig. 2E and F), and BrdU staining also revealed a reduced DNA synthesis rate in IDH1-mutant glioma cells (Fig. 2F). The immunofluorescence (IF) staining results for γ-H2A.X, along with increased XPD and POLD1 expression, further revealed enhanced DNA damage in IDH1-mutant U87 cells (Fig. 2G and H). These results suggest that IDH1 mutations promote cellular senescence in glioma cells by increasing ROS production and DNA damage.

Furthermore, we investigated whether D-2-hydroxyglutarate (D-2HG), an oncometabolite in IDH-mutant tumors, contributes to cell senescence in IDH-mutant glioma cells. After treatment with 10 mM D-2HG for 72 hours, SA-β-gal staining revealed that the senescence of both U87 and U251 glioma cells was significantly accelerated (Fig. 2I and J and Supplementary Fig. 1A and B). In U87 cells, D-2HG exposure also consistently increased ROS production (Fig. 2K), decreased NADPH production (Fig. 2L) and elevated the expression of DNA repair markers, including XPD and POLD1 (Fig. 2M). Fluorescence staining further revealed increased γ-H2A.X expression and reduced BrdU incorporation in D-2HG-treated U87 cells, indicating enhanced DNA damage and disrupted DNA synthesis (Fig. 2N). Collectively, these findings suggest that D-2HG promotes senescence in IDH-mutant glioma cells by inducing ROS production and DNA damage.

### Glutamine Alleviates the Senescence of IDH1-mutant Glioma Cells

**Figure 3.**
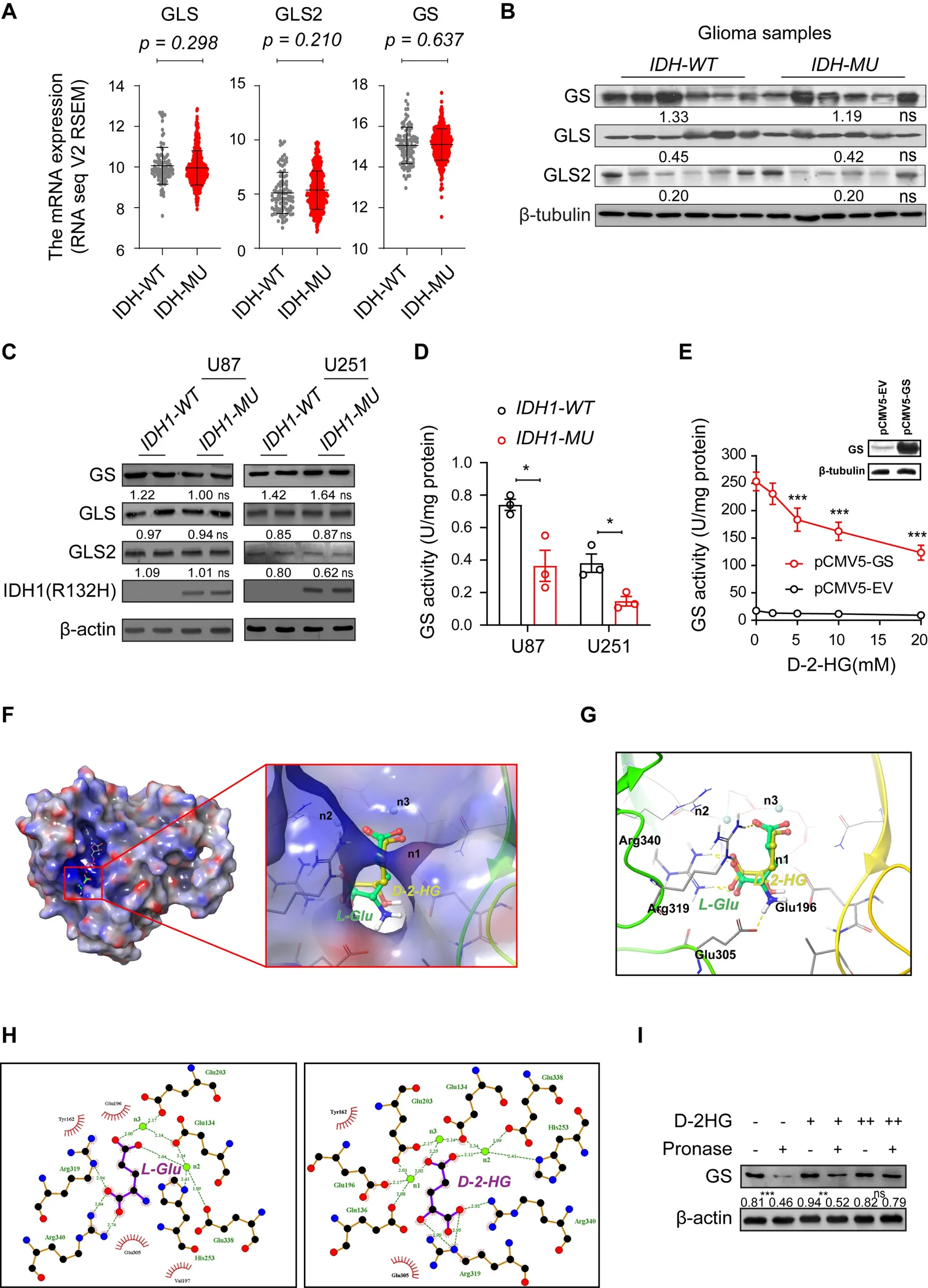
**Glutamine alleviates the senescence of IDH1-mutant glioma cells**. (A&B) Growth curves of *IDH1-WT* and *IDH1-MU* U87 (A) and U251 (B) cells in the presence of 0.2 mM (low) or 4 mM (high) glutamine (Gln), n = 4 per group. (C&D) SA-β-gal staining (C) and representative immunoblotting of p53, p21 and p16 (D) of *IDH1-WT* and *IDH1-MU* U87 and U251 glioma cells cultured with low and high levels of Gln. Scale bar, 100 μm. n = 6 per group in C, and represents of three individual experiments in D. (E&F) SA-β-gal staining (E) and representative immunoblotting of p53, p21 and p16 (F) in *IDH1-WT* and *IDH1-MU* U87 and U251 glioma cells treated with 10 mM D-2HG in the presence of 0.2 mM (low) or 4 mM (high) of Gln. Scale bar, 100 μm. n = 6 per group in E and representative of three individual experiments in F. Data are presented as percentages of SA-β-gal-positive cells after normalization to the total cell number for each group (C, E). β-tubulin or β-actin was used for immunoblot normalization. All the data are presented as the mean ± SEMs, and statistical significance was determined using unpaired Student’s t test (A and B) and one-way ANOVA (C, D, E, F) (*p < 0.05, **p < 0.01, ***p < 0.001).

Glutamine is crucial for the survival of senescent cells [20], and previous research has shown that IDH1 mutations significantly reduce glutamine levels in both astrocytes and glioma cells [4, 25]. We observed that the growth of IDH1-mutant U87 and U251 cells was markedly inhibited under culture conditions with low levels of glutamine (0.2 mM) (Fig. 3A and B). Additionally, SA-β-gal staining revealed that high levels of glutamine (4 mM) significantly alleviated the senescence of IDH1-mutant U87 and U251 glioma cells (Fig. 3C). Immunoblot analysis confirmed that glutamine reduced the expression of the senescence markers p53, p21, and p16 in these cells (Fig. 3D). Given that D-2HG is a major driver of senescence in IDH1-mutant cells, our data further indicated that high glutamine levels decreased SA-β-gal-positive cells and the expression of p53, p21 and p16 in D-2HG-treated U87 and U251 cells (Fig. 3E and F), suggesting that glutamine can mitigate senescence in IDH1-mutant cells.

To investigate whether reduced endogenous glutamine contributes to IDH mutation-induced cellular senescence, we treated wild-type IDH cells with the glutamine synthetase (GS) inhibitor L-methionine-DL-sulfoximine (MSO). MSO treatment was found to induce cellular senescence (Supplementary Fig. 2A and B), promote DNA damage (Supplementary Fig. 2C), increase intracellular ROS levels (Supplementary Fig. 2D), and decrease NADPH production (Supplementary Fig. 2E). These findings indicate that the inhibition of GS activity facilitates the onset of cellular senescence.

### D-2HG Competitively Inhibits GS Activity in Glioma Cells

**Figure 4.**
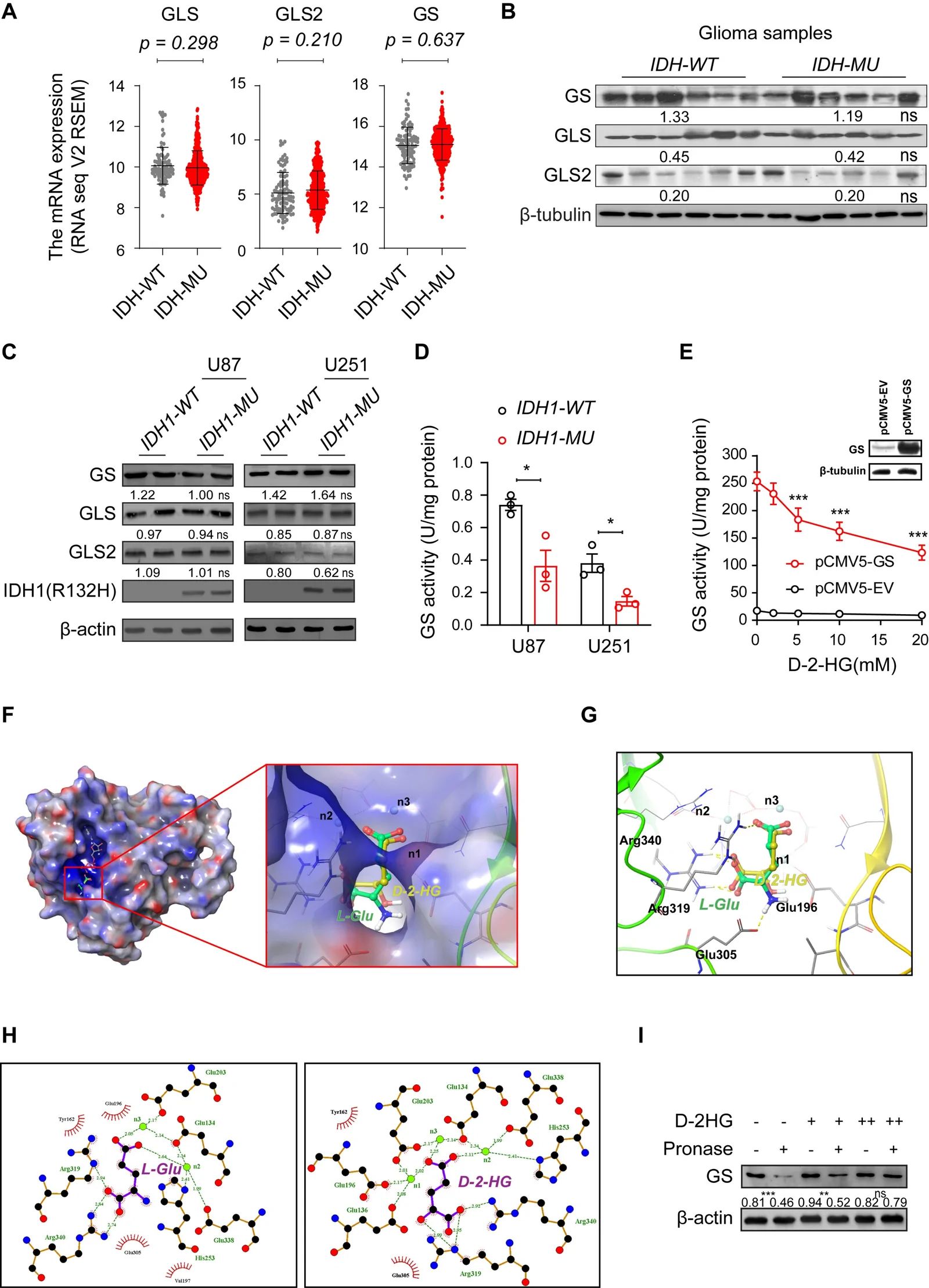
**D-2HG competitively inhibits GS activity in glioma cells.(A) The mRNA levels of GS, GLS2 and GLS in IDH-wildtype (*IDH-WT*) and IDH-mutant (*IDH-MU*) LGGs were analyzed according to TCGA data (n = 96 in the *IDH-WT* group, n = 415 in the *IDH-MU* group)**. (B&C) Representative immunoblotting of GS, GLS2 and GLS levels in *IDH-WT* and *IDH-MU* glioma tissues (B) and IDH1-mutant U87 and U251 glioma cells (C). n = 6 per group in B and n = 2 per group in C. (**D**) GS activity assay in *IDH1-WT* and *IDH1-MU* U87 and U251 cells, n = 3. (**E**) GS activity in GS-overexpressing 293T cells after treatment with D-2HG; n = 5. (F&G) The substrate-binding site (F) and H-bond formation (G) of D-2-HG (yellow carbons) and L-Glu (green carbons) in GS protein. (**H**) Glu interacts with protein side chains as well as two of the three Mn^2+^ ions in the active site (those designated n2 and n3, in accordance with earlier conventions). (**I**) Drug affinity response target stability (DARTS) showing the protection of D-2HG against the pronase degradation of GS; the data are representative of three individual experiments. β-tubulin or β-actin was used for immunoblot normalization. All the data are presented as the means ± SEMs, and statistical significance was determined using unpaired Student’s t tests (B, D and E) and Mann-Whitney tests (C) (*p < 0.05, **p < 0.01, ***p < 0.001).

Although glutamine synthetase plays a critical role in IDH1-mutant glioma cell senescence, data from The Cancer Genome Atlas (TCGA) indicated that the mRNA levels of glutamine synthetase (GS), glutaminase (GLS), and glutaminase 2 (GLS2) were comparable between IDH-mutant and IDH-wildtype gliomas (Fig. 4A). Our immunoblot analysis also confirmed these unchanged GS, GLS and GLS2 levels in both IDH-mutant glioma tissues and IDH1-mutant glioma cells (Fig. 4B and C). Notably, our results revealed that GS enzyme activity was significantly reduced in IDH1-mutant U87 and U251 glioma cells (Fig. 4D). In GS-overexpressing 293T cells, D-2HG inhibited GS activity in a dose-dependent manner (Fig. 4E). Moreover, structural analysis of the GS protein and its glutamine (L-Glu) binding site using AutoDock revealed that D-2HG, owing to its structural similarity to L-Glu, fits readily into the L-Glu binding site (Fig. 4F). The binding affinities (ΔG_Bind_) for L-Glu and D-2HG with GS were calculated to be -10.51 and -9.49 kcal/mol, respectively. A more negative value indicates stronger binding of the ligand to the target protein. Superimposition analysis revealed that the Arg319 and Arg340 residues of GS interact similarly with the carboxyl group at the first position of both D-2HG and L-Glu (Fig. 4G). Furthermore, each polar atom of D-2HG establishes multiple interactions with the GS protein either directly or through metal-mediated contacts involving all three Mn2+ ions (Fig. 4H). Drug affinity response target stability (DARTS) assays also demonstrated that D-2HG protected GS from pronase degradation, confirming the direct binding of D-2HG to GS (Fig. 4I). Collectively, these findings suggest that D-2HG competitively binds to the L-Glu-binding sites of GS and inhibits GS activity.

**Figure 5.**
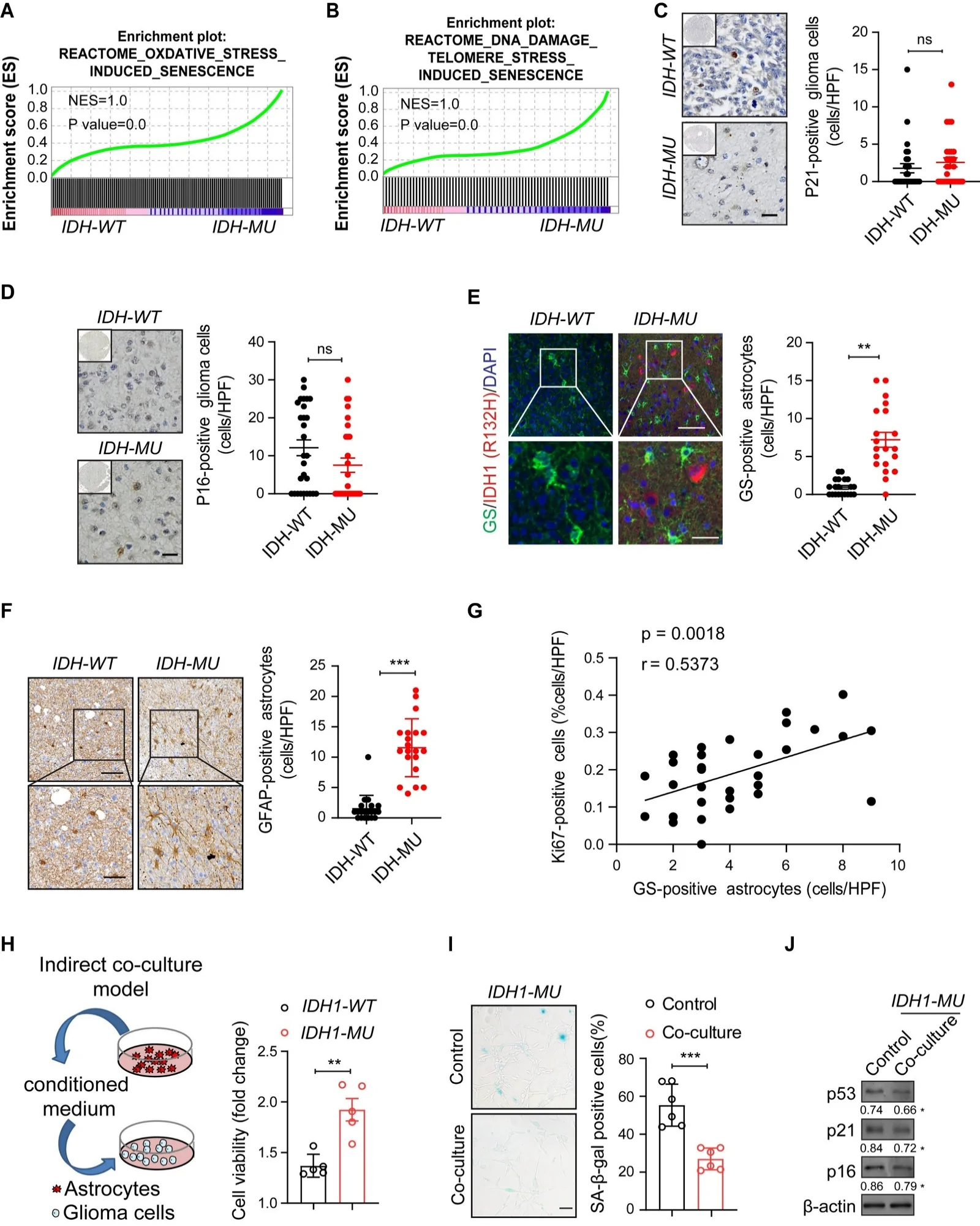
**Astrocytes alleviate the senescence of glioma cells in IDH-mutant glioma tissues**. (A&B) Reactomes of the oxidative stress-induced senescence (A) and DNA damage/telomere stress-induced senescence (B) in *IDH-WT* and *IDH-MU* glioma samples from TCGA data. (C&D) Representative IHC staining and statistical analysis of p21 (C) and p16 (D) in *IDH-WT* and *IDH-MU* glioma tissues. n = 28 per group in C and D. (**E**) IF staining of GS (green) and IDH1(R132H) (red) in *IDH-WT* and *IDH-MU* glioma tissues, with the nuclei stained with DAPI (blue). Scale bars are 50 μm (top) and 20 μm (bottom), n = 20 per group. (**F**) IHC staining and statistical analysis of GFAP in IDH-WT and IDH-MU glioma tissues. Scale bars are 50 μm (top) and 20 μm (bottom), n = 20 per group. (**G**) The correlation between the Ki67 index of glioma cells and the percentage of GS-positive astrocytes in *IDH-MU* glioma (n = 31, Pearson correlation coefficient, two-tailed r = 0.5373, p = 0.0018). (**H**) Indirect co-culture of primary astrocytes and *IDH1-MU* glioma cells (left) and the growth of *IDH1-MU* U87 cells in medium from astrocytes (right) (n = 5 per group). The total number of cells after co-culture for 3 days was normalized to the initial seeded cell number. (**I**) SA-β-gal staining of *IDH1-MU* U87 cells under co-culture conditions. Scale bar, 100 μm; n = 6 per group. The data are presented as percentages of SA-β-gal-positive cells after normalization to the total number of cells in each group. (**J**) Immunoblotting for p53, p21 and p16 in *IDH1-MU* U87 cells co-cultured with astrocytes; the data are representative of three individual experiments. β-actin was used for normalization. All the data are presented as the means ± SEMs, and statistical significance was determined via unpaired Student’s t tests (C, D, E, F, H, I and J) (*p < 0.05, **p < 0.01, ***p < 0.001; ns, not significant).

### Glioma-associated Astrocytes (GAAs) Alleviate Cell Senescence in IDH-mutant Glioma Tissues by Supplying Adequate Glutamine

We further assessed cellular senescence in IDH-mutant glioma tissues. Gene set enrichment analysis (GSEA) of the TCGA database revealed active DNA damage and ROS-induced senescence signaling pathways in both IDH-wildtype and IDH-mutant glioma tissues (Fig. 5A and B). Immunohistochemistry (IHC) confirmed comparable expression levels of p16 and p21 in IDH-wildtype and IDH-mutant tissues (Fig. 5C and D). Interestingly, IF staining revealed numerous GS-positive cells in IDH1-mutant glioma tissues (Fig. 5E), which were characterized as glioma-associated astrocytes (GAAs). GFAP staining further revealed an increased presence of the classically branched GAAs in IDH-mutant glioma tissues (Fig. 5F). GAAs are known to support glioma cell proliferation by providing adequate glutamine [32, 33]. Using an indirect co-culture assay, we found that astrocytes significantly promoted the proliferation of IDH1-mutant U87 glioma cells (Fig. 5H) and reduced the number of SA-β-gal-positive cells (Fig. 5I). Additionally, co-culture with astrocytes decreased the expression of senescence markers, including p53, p21, and p16, in IDH-mutant U87 cells (Fig. 5J), suggesting that GAAs mitigate senescence in IDH-mutant glioma cells *in vivo*. Collectively, these findings indicate that GS-positive GAAs alleviate cellular senescence in IDH-mutant gliomas by providing adequate glutamine.

### Blocking Glutamine Transport in Astrocytes Promotes Cell Senescence and Inhibits the Growth of IDH-mutant Glioma Cells

**Figure 6.**
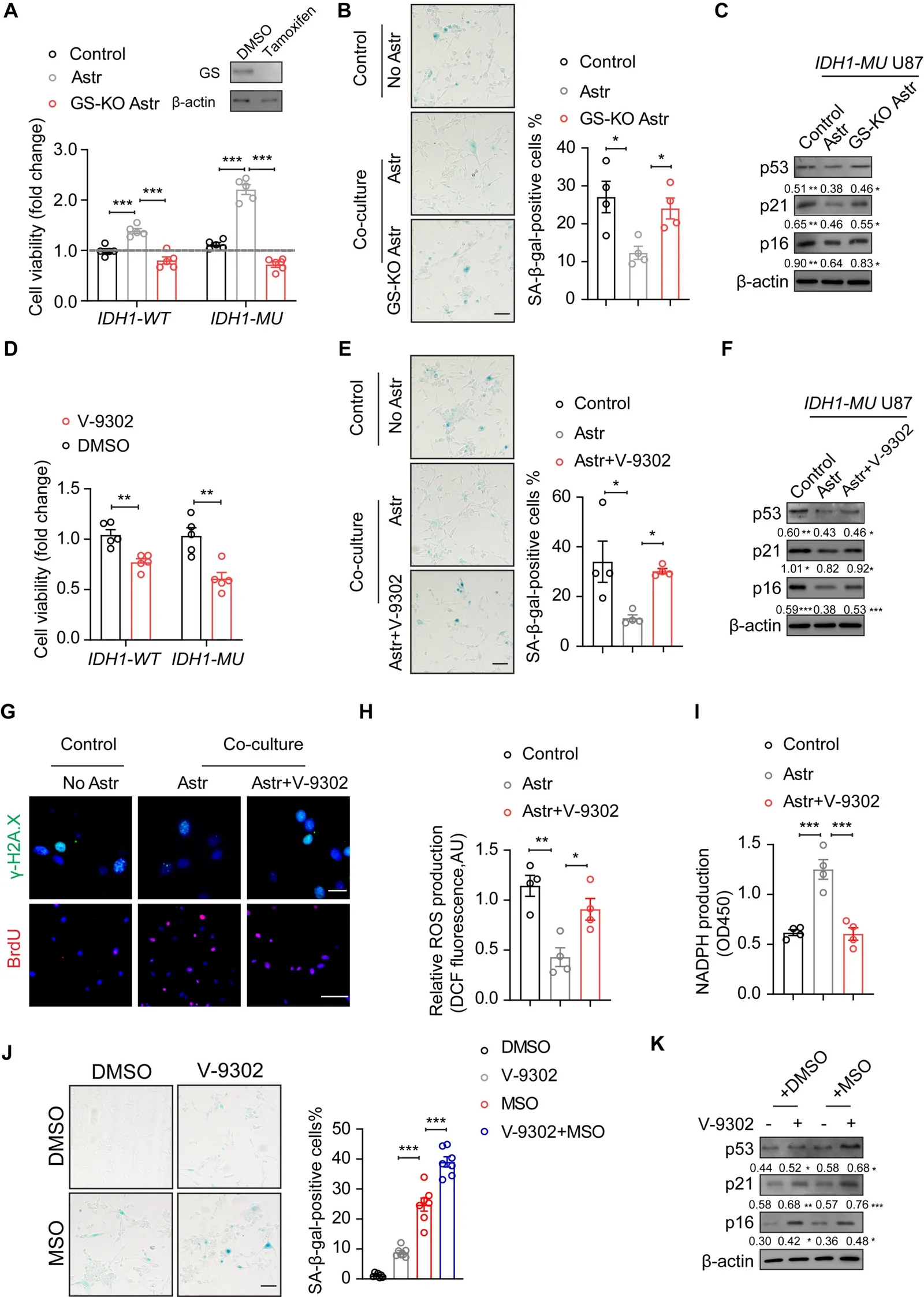
**Blocking Gln transport promotes the cell senescence and inhibits the growth of IDH-mutant glioma cells**. (**A**) Growth of *IDH1-WT* and *IDH1-MU* U87 glioma cells after co-culture with wild-type (*GS^+/+^*) and GS knockout (*GS^-/-^*) astrocytes (Astr). The data are representative of three individual experiments with n = 5 per group (normalization: control). (B&C) SA-β-gal staining (B) and immunoblotting for p53, p21 and p16 (C) in *IDH1-MU* U87 cells after co-cultured with *GS*^+/+^ or *GS*^-/-^ astrocytes. Scale bar, 100 μm. n = 4 per group in B and representative of three individual experiments in C. (**D**) Growth of *IDH1-WT* and *IDH1-MU* U87 cells after co-cultured with astrocytes in the presence of 5 μM V-9302 (SLC1A5 inhibitor). The data are representative of three individual experiments with n = 5 per group. (E&F) SA-β-gal staining (E) and immunoblotting for p53, p21 and p16 (F) in *IDH1-MU* U87 glioma cells co-cultured with astrocytes in the presence of V-9302; scale bar, 100 μm. n = 4 per group in E and representative of three individual experiments in F. (**G-I**) Immunofluorescence staining of γ-H2A.X (G, upper), BrdU (G, bottom), relative intracellular ROS (H) and NADPH production (I) in *IDH1-MU* U87 cells co-cultured with astrocytes and V-9302. Nuclei were counterstained with DAPI (blue). Scale bars are 20 μm (upper) and 100 μm (bottom). Data are representative of three individual experiments in G, n = 4 per group in H. (J&K) SA-β-gal staining (J) and immunoblotting for p53, p21 and p16 (K) in *IDH1-WT* U87 glioma cells co-cultured with astrocytes in the presence of MSO and V-9302. Scale bar, 100 μm. Data are presented as a percentage of SA-β-gal-positive cells to the total cell number in each group (B, E, J), and β-actin was used for immunoblot normalization. All the data are presented as means ± SEMs, and statistical significance was determined using one-way ANOVA (A, B, C, D, E, F, H, I, J, K) and the Kruskal–Wallis tes (K). (*p < 0.05, **p < 0.01, ***p < 0.001).

**Figure 7.**
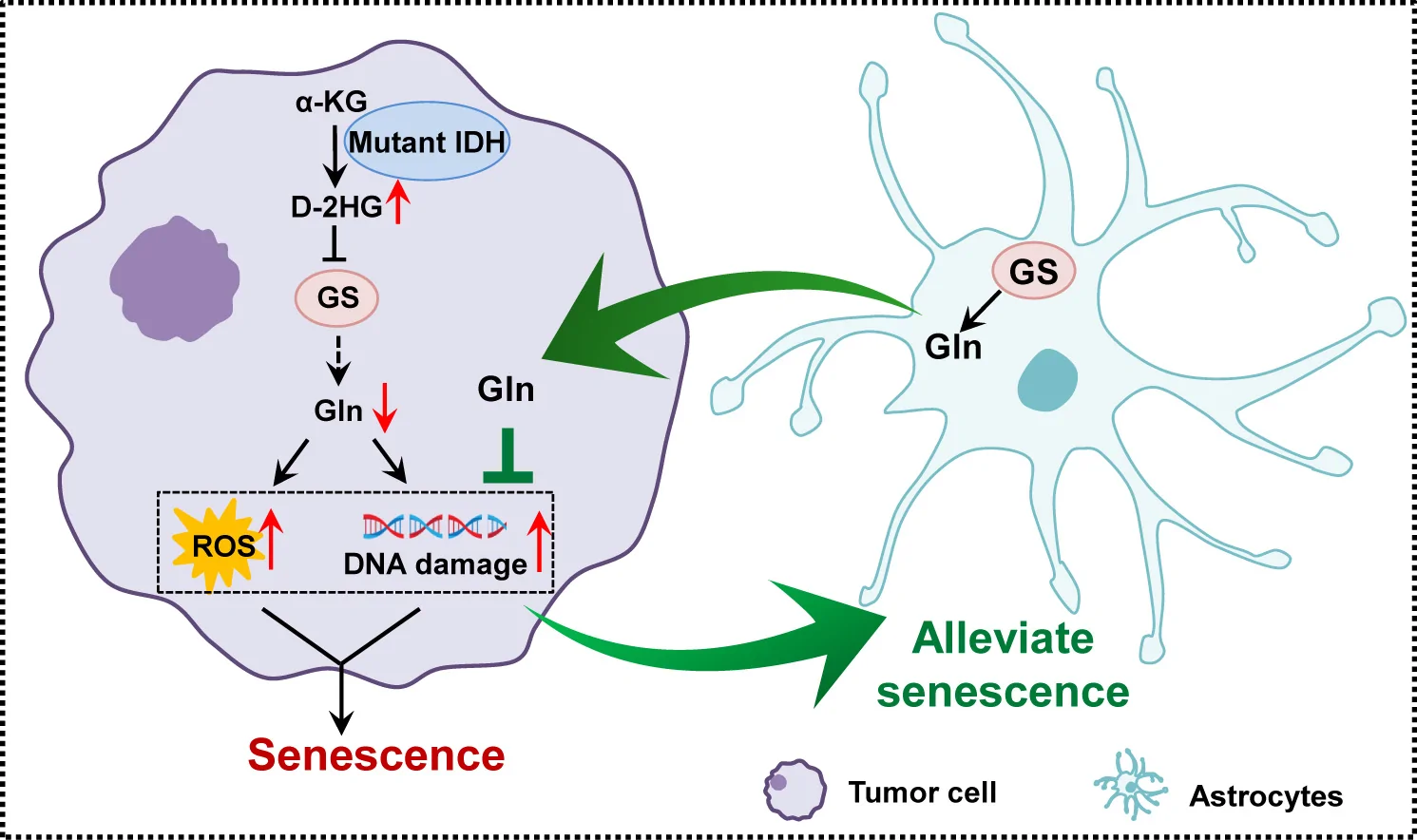
**Schematic diagram showing that D-2HG reduces endogenous glutamine production by inhibiting GS activity, resulting in the senescence of IDH1-mutant glioma cells**. Astrocytes are able to alleviate senescence by providing adequate glutamine to glioma cells.

To explore whether glioma-associated astrocytes (GAAs) mitigate the senescence of IDH-mutant glioma cells, we co-cultured these cells with GS-knockout (GS^-/-^) astrocytes. Our results indicated that GS-knockout astrocytes failed to improve the proliferation of IDH-mutant U87 glioma cells *in vitro* (Fig. 6A) and were not able to alleviate the senescence of IDH1-mutant glioma cells (Fig. 6B and C). To further assess the involvement of glutamine transport, we utilized V-9302, an SLC1A5 inhibitor, to block glutamine exchange between astrocytes and glioma cells. V-9302 treatment significantly reduced the proliferative effects of astrocytes on IDH-mutant U87 cells (Fig. 6D) and diminished their capacity to alleviate cellular senescence (Fig. 6E and F). Additionally, astrocyte co-culture slowed DNA damage in IDH-mutant U87 cells, promoted DNA synthesis (Fig. 6G), reduced the accumulation of ROS (Fig. 6H), and increased NADPH production (Fig. 6I). Notably, these effects were abrogated upon the administration of V-9302 (Fig. 6G-I). In IDH wild-type U87 cells, we further assessed the effects of MSO and V-9302, and the results revealed that V-9302 potentiated the proaging effects of MSO (Fig. 6J and K). Additionally, *in vivo* results indicated that V-9302 more significantly inhibited the tumor growth inhibition of IDH-mutant gliomas compared to IDH wild-type gliomas (Supplementary Fig. 3A). Collectively, our findings suggest that D-2HG competitively inhibits GS activity, reducing endogenous glutamine production and resulting in cellular senescence by increasing DNA damage and ROS accumulation. GS-enriched GAAs can supply adequate glutamine, counteracting the senescence of IDH1-mutant glioma cells (Fig. 7). Moreover, blocking glutamine transport from GAAs to glioma cells promotes senescence and inhibits the growth of IDH-mutant gliomas, highlighting a potential therapeutic approach for IDH1-mutant gliomas.

## DISCUSSION

IDH mutations commonly arise early in gliomagenesis in lower-grade gliomas, where they are frequently accompanied by other proto-oncogene amplifications or tumor suppressor gene deletions [34, 35]. D-2HG, produced by mutant IDH enzymes, has been shown to induce epigenetic alterations and metabolic reprogramming in tumors. Despite these oncogenic effects, patients with IDH-mutant gliomas generally have better prognoses than their IDH-wildtype counterparts do. These observations underscore the need for further investigation into the specific mechanisms underlying the unique biological and clinical behaviors of IDH-mutant gliomas.

In this study, astrocyte-specific mutant IDH1 knock-in mice developed severe hydrocephalus and a marked glial cell senescence phenotype in astrocytes. More importantly, we found that IDH1 mutation induced senescence in glioma cells by increasing ROS production and significant DNA damage. Various stressors can induce cellular senescence, with nuclear DNA damage frequently cited as a fundamental cause [36, 37]. Persistent DNA damage leads to sustained DNA damage response (DDR) signaling and prolonged proliferative arrest, resulting in cellular senescence [38, 39]. ROS are also considered key initiators of senescence because of their role in amplifying oxidative stress, as telomeres are particularly susceptible to this oxidative damage [40]. Additionally, ROS production exacerbates DNA damage and DDR signaling, which are also essential for the commitment to senescence [40].

IDH mutations have been previously reported to increase DNA damage [6, 7], in which D-2HG plays a significant role. Research has shown that D-2HG production depletes NADPH and impedes glutathione synthesis in IDH-mutant cancers, subsequently increasing ROS levels[2, 41]. D-2HG also reduces the activity of branched-chain amino acid transaminase (BCAT) and lowers glutamate levels, potentially diminishing glutathione synthesis and exacerbating cellular oxidative stress [4]. In this study, our results indicated that D-2HG induced senescence in IDH1-mutant glioma cells by increasing both ROS production and DNA damage.

Metabolism is fundamental to all cellular processes, including cell genesis, survival, homeostasis, proliferation, aging, and cell death, as it supplies both energy and essential substrates. The study of metabolic alterations in cancer has identified critical energy pathways that drive tumor growth and survival, revealing potential therapeutic targets. Furthermore, numerous treatments targeting tumor metabolic pathways are actively being developed and have demonstrated promising therapeutic outcomes [42, 43]. Previous research has shown that glutaminolysis is essential for the survival of senescent cells [20]. Here, we found that exogenous glutamine significantly mitigated cellular aging and decreased the proliferation of IDH1-mutant astrocytes and glioma cells. Genetic and pharmacological inhibition of glutaminase 1 (GLS1) can sensitize senescent cells, alleviating several aging phenotypes associated with senescence [20]. Recent findings further highlight that inhibiting glutaminolysis can abrogate the mitochondrial dysfunction that is commonly observed in senescent cells [44]. Recent studies have demonstrated that inhibiting glutamine catabolism markedly suppresses glioma growth [45-47].Notably, GLS inhibitors exert pronounced lethal effects on IDH-mutant cells [48]. In IDH-mutant cells, D-2-HG has been shown to inhibit BCAT, increasing glutamine dependence and sensitivity to glutaminase inhibition in glioma cells [4].

GS is the only enzyme responsible for glutamine synthesis, and its expression varies widely across different tumor types, underscoring the adaptability of glutamine metabolism in tumor cells [49, 50]. Tumors with lower GS expression often exhibit increased levels of glutamine transporters, which is correlated with an increased dependence on extracellular glutamine [49]. These cells frequently utilize glutamine secreted by neighboring cells in the tumor microenvironment, including astrocytes, stromal cells, adipocytes, and macrophages, which typically express high GS levels [51-53]. This intercellular interaction can significantly impact tumor cell proliferation and metastasis. In many tumors, GS primarily synthesizes glutamine for nucleic acid biosynthesis rather than fueling the tricarboxylic acid (TCA) cycle, thereby promoting cell proliferation, as observed in glioblastoma multiforme (GBM) and liver cancers [32, 54]. Additionally, GS knockdown or inhibition has been shown to impair tumor growth and progression in breast and ovarian cancers [55-57]. Recent research has shown that GS, which is a powerful antioxidant, facilitates nucleotide synthesis, which is essential for efficient DNA repair, and elevates the levels of glutathione [58, 59]. In this study, we observed that GS inhibition diminishes endogenous glutamine synthesis and induces senescence in glioma cells. Notably, we discovered that D-2-hydroxyglutarate (D-2HG) inhibits glutamine synthesis by competing with glutamate binding sites in GS. Inaddition, GS is subject to multiple forms of post-translational modifications, including acetylation, ubiquitination and palmitoylation [60-62]. These modifications are closely related to the expression, localization and function of GS, and deserve further exploration in glioma research.

However, characteristics of cellular senescence are not observed in IDH-mutant glioma tissues. In the brain, astrocytes with high GS expression provide sufficient glutamine to support neuronal function [21]. Our study revealed an increased number of glioma-associated astrocytes (GAAs) in IDH-mutant glioma tissues, which are able to promote the proliferation of IDH-mutant glioma and reduce senescence-related characteristics by supplying adequate glutamine. Recent studies have shown that nicotinamide induces reversible senescence in glioblastoma by downregulating SOX2, nicotinamide N-methyltransferase (NNMT), and nicotinamide phosphoribosyltransferase (NAMPT), thus disrupting critical pathways involved in cell identity and metabolic regulation [63]. Similarly, our findings indicate that D-2HG-induced senescence is reversible (Supplementary Fig. 4A and B). Additionally, exogenous glutamine alleviates the cellular senescence caused by D-2HG. IDH-mutant glioma cells continuously produce D-2HG, sustaining its prosenescent effects. Thus, limiting exogenous glutamine uptake may serve as a promising therapeutic strategy to increase senescence in these cells. Previous *in vivo* studies have shown that simultaneous inhibition of the glutamine transporter SLC1A5 and lipid metabolism significantly suppresses glioma proliferation [64]. In line with these findings, our results revealed that V-9302 markedly induced cellular senescence in IDH1-mutant glioma cells. Notably, V-9302 treatment exhibited greater inhibitory effects on tumor growth in IDH1-mutant gliomas compared to the wild-type groups, both in vivo and ex vivo. In summary, our findings demonstrate that D-2HG acts as a competitive inhibitor of glutamine synthetase, thereby reducing endogenous glutamine synthesis. This reduction in glutamine synthesis increases DNA damage and ROS production, which collectively promote cellular senescence phenotypes. Additionally, our study suggests that targeting glutamine transporters with specific inhibitors might be a promising therapeutic strategy for IDH-mutant glioma.

Epigenetic modifications are recognized as markers of cellular aging, manifesting as increased H3K4 trimethylation, decreased H3K9 and H3K27 trimethylation, and global DNA hypomethylation. Nonetheless, definitive evidence linking these epigenetic patterns to the direct causation of aging remains inconclusive [65]. It is well established that D-2HG competitively inhibits α-kg-dependent dioxygenases, including histone demethylases and TET DNA demethylases, thereby inducing widespread hypermethylation, including elevated levels of H3K27, H3K4, and DNA methylation [3, 66], such changes may contribute to cellular aging indirectly through alternative signaling pathways [67]. Based on our findings, we propose that the senescence phenotype observed in IDH-mutant cells is not primarily attributable to these epigenetic alterations. In this investigation, glutamine deficiency emerged as one factor contributing to the cellular senescence induced by IDH mutations, but glutamine supplementation alone was insufficient to fully counteract the aging phenotypes associated with these mutations, indicating the involvement of additional mechanism. Moreover, IDH-mutant glioma tissues frequently exhibit TP53 mutations, and the loss of genetic alterations in PTEN is closely associated with cellular aging [34, 35]. The specific relationship between IDH mutations and these key oncogenic changes in the context of tumor cell senescence remains to be elucidated.

## Supplementary Materials

The Supplementary data can be found online at: www.aginganddisease.org/EN/10.14336/AD.2025.0554.



## Data Availability

The GraphPad prism data and the sources of the GraphPad prism data are available in Supplementary Data 1 & 2. The TCGA data are shown in Supplementary Data 3. All other data described, analyzed and represented in the figures presented in this study are available from the corresponding authors upon reasonable request.
