# Supplementary material for: D-2HG Inhibits Glutamine Synthetase Activity and Induces Senescence in IDH-Mutant Gliomas
Source: Aging Dis. 2026 Aug 4;17(5):2636–53. doi: 10.14336/AD.2025.0554 (PMC13437120; doi:10.14336/AD.2025.0554)
Supplement: Supplementary file 1 [file AD-17-5-2636-s.pdf]

SUPPLEMENTARY DATA

D-2HG Inhibits Glutamine Synthetase Activity and Induces Senescence in IDH-Mutant Gliomas

Yuanlin Zhao, Jiankuan Shi, Yuan Yuan, Ying Yang, Xing Gao, Risheng Yang, Yuying Wang, Lijun Zhang, Jing Li, Peizhen Hu, Yingmei Wang, Chao Sun, Ruili Han, Yuqiao Xu, Zengshan Li, Zhe Wang, Yu Gu, Jing Ye

© 2025. Zhao Y et al. Published online at http://www.aginganddisease.org/EN/10.14336/AD.2025.0554

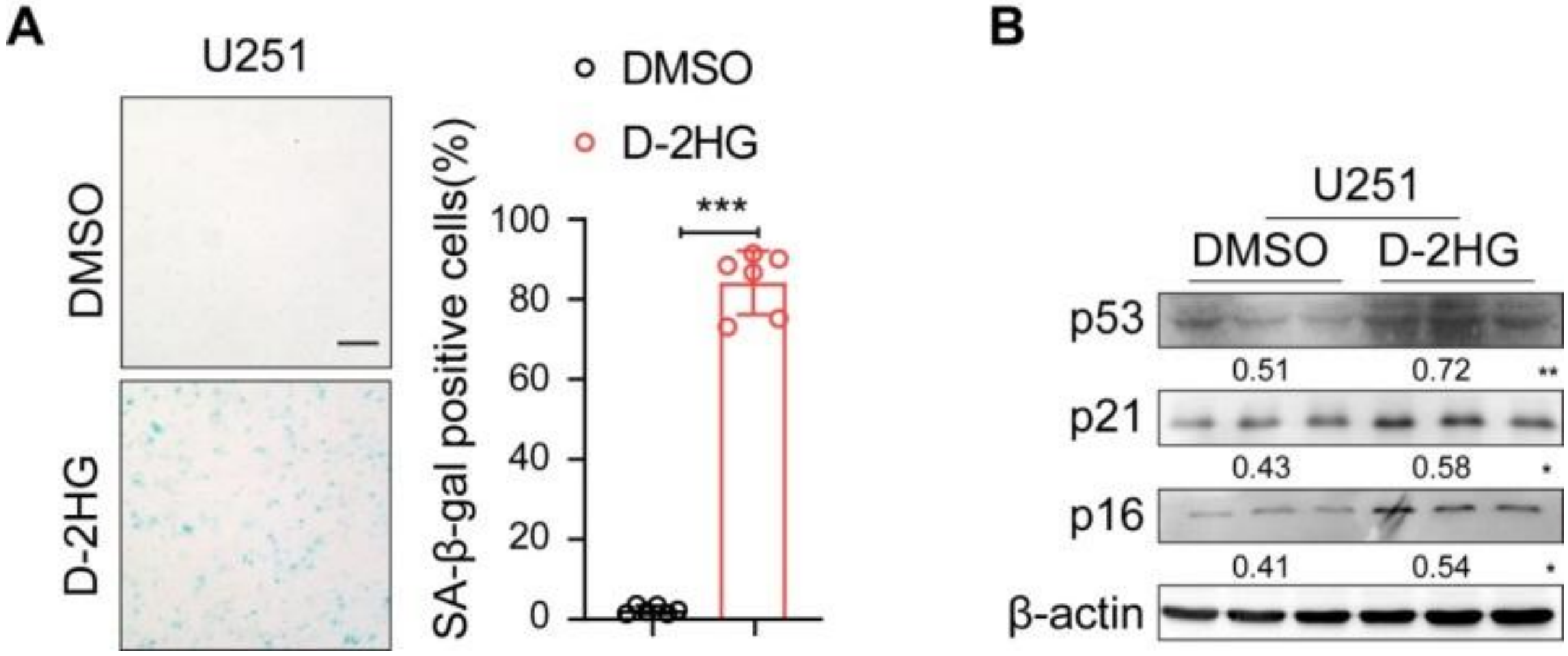

Supplementary Figure 1. D-2HG induces the senescence in U251 cells. (**A**) SA-β-gal staining of U251 glioma cells after treatment with 10 mM D-2HG for 72 hr. Scale bar, 100 μm. (**B**) Immunoblotting of p53, p21 and p16 in U251 glioma cells after treatment with 10 mM D-2HG. The data are presented as percentages of SA-β-gal-positive cells to the total number of cells in each group, and β-actin was used for immunoblot normalization. Data are representative of three individual experiments with n = 6 per group in A and n = 3 per group in B. All data are presented as the means ± SEMs, and statistical significance was determined using unpaired Student's t tests (A and B). (*p < 0.05, **p < 0.01, ***p < 0.001).

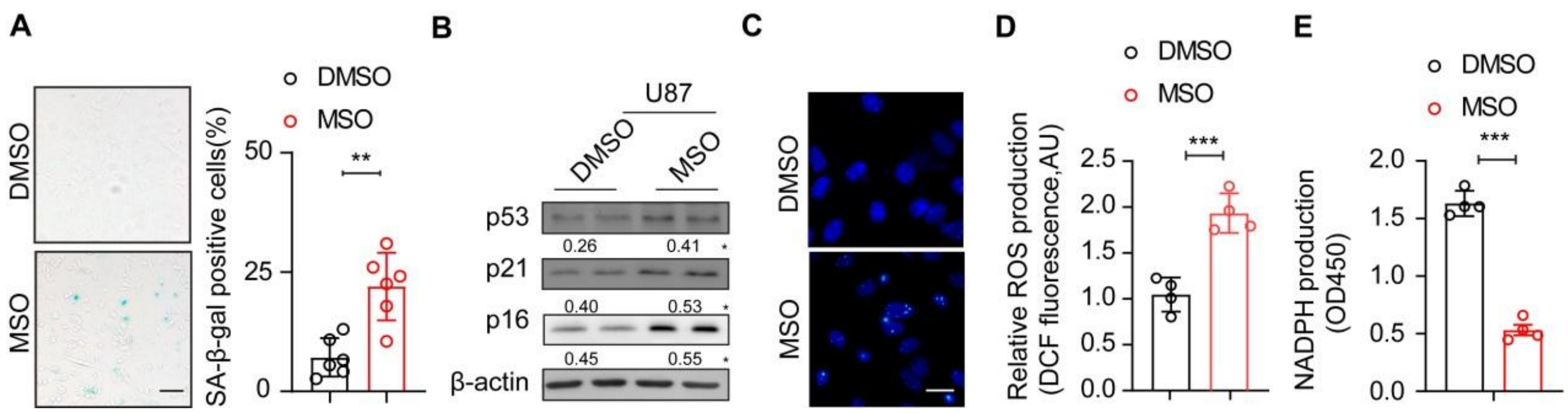

Supplementary Figure 2. GS inhibitor (MSO) induces the cell senescence in U87 cells. (**A-E**) SA-β-gal staining (**A**), immunoblotting of p53, p21 and p16 (**B**), and immunofluorescence staining of γ-H2A.X (**C**), relative intracellular ROS generation (normalization: DMSO) (D) and NADPH production in *IDH1-WT* U87 glioma cells cultured with 5 mM MSO (n = 6 in **A**, n = 3 in **B&D**, n = 4 in **C**). Scale bars are 100 μm (**A**) and 20 μm (**C**). The data are presented as a percentage of SA-β-gal-positive cells to the total cell number in each group, β-actin was used for immunoblot normalization. All the data are presented as means ± SEMs, and statistical significance was determined using unpaired Student's t tests (A, B, D, E) and Mann–Whitney tests (B) . (*p < 0.05, **p < 0.01, ***p < 0.001).

© 2025. Zhao Y et al. Published online at http://www.aginganddisease.org/EN/10.14336/AD.2025.0554

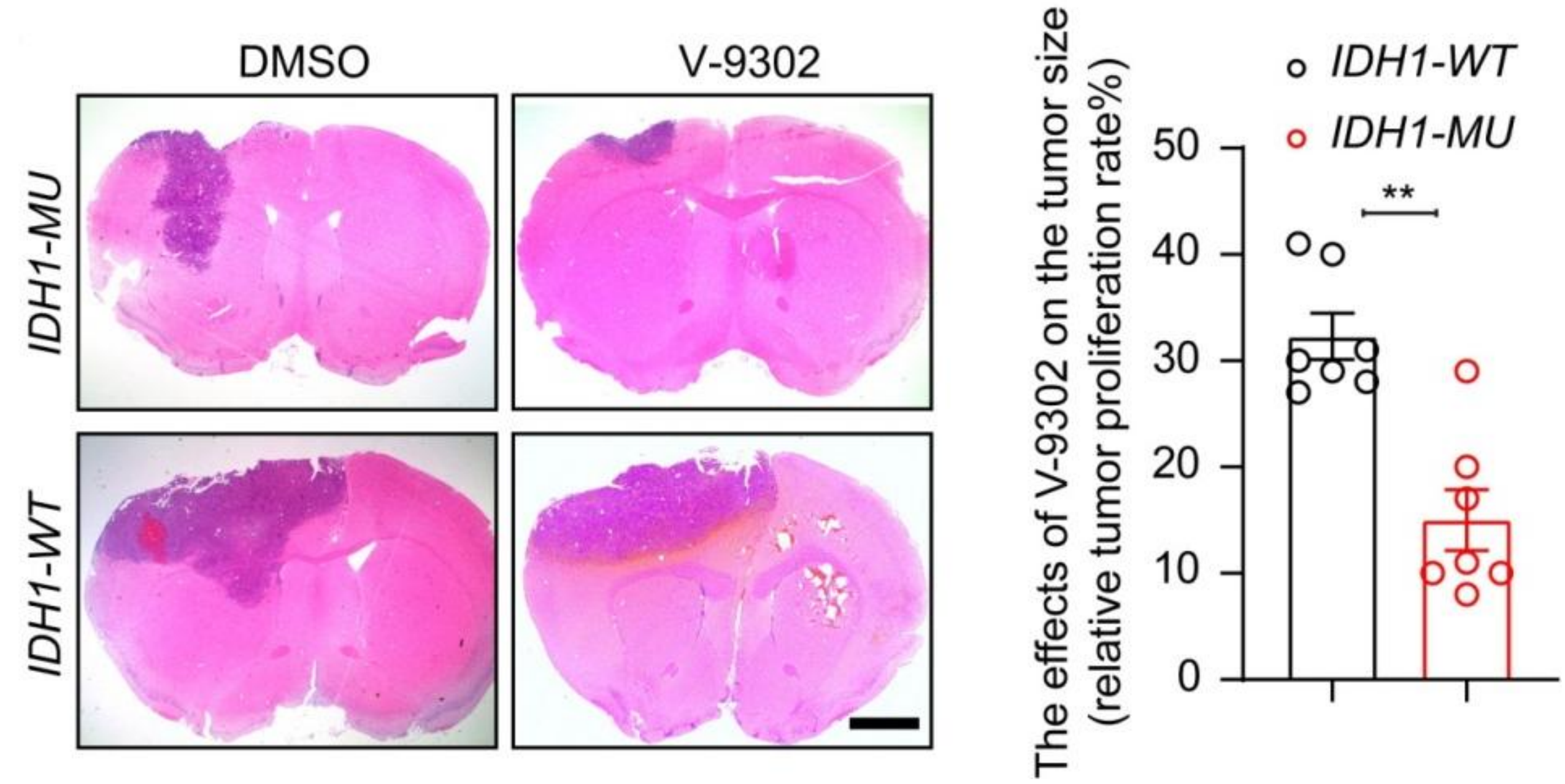

Supplementary Figure 3. The anticancer efficacy of V-9302 in orthotopic xenograft glioma mice. Representative images (left) and relative tumor proliferation statistical analysis (normalization: DMSO) (right) of H&E staining showing tumor volume in the nude mice, Scale bar, 1 mm. All the data are presented as means ± SEMs, and statistical significance was determined using Mann–Whitney tests (*p < 0.05, **p < 0.01, ***p < 0.001)

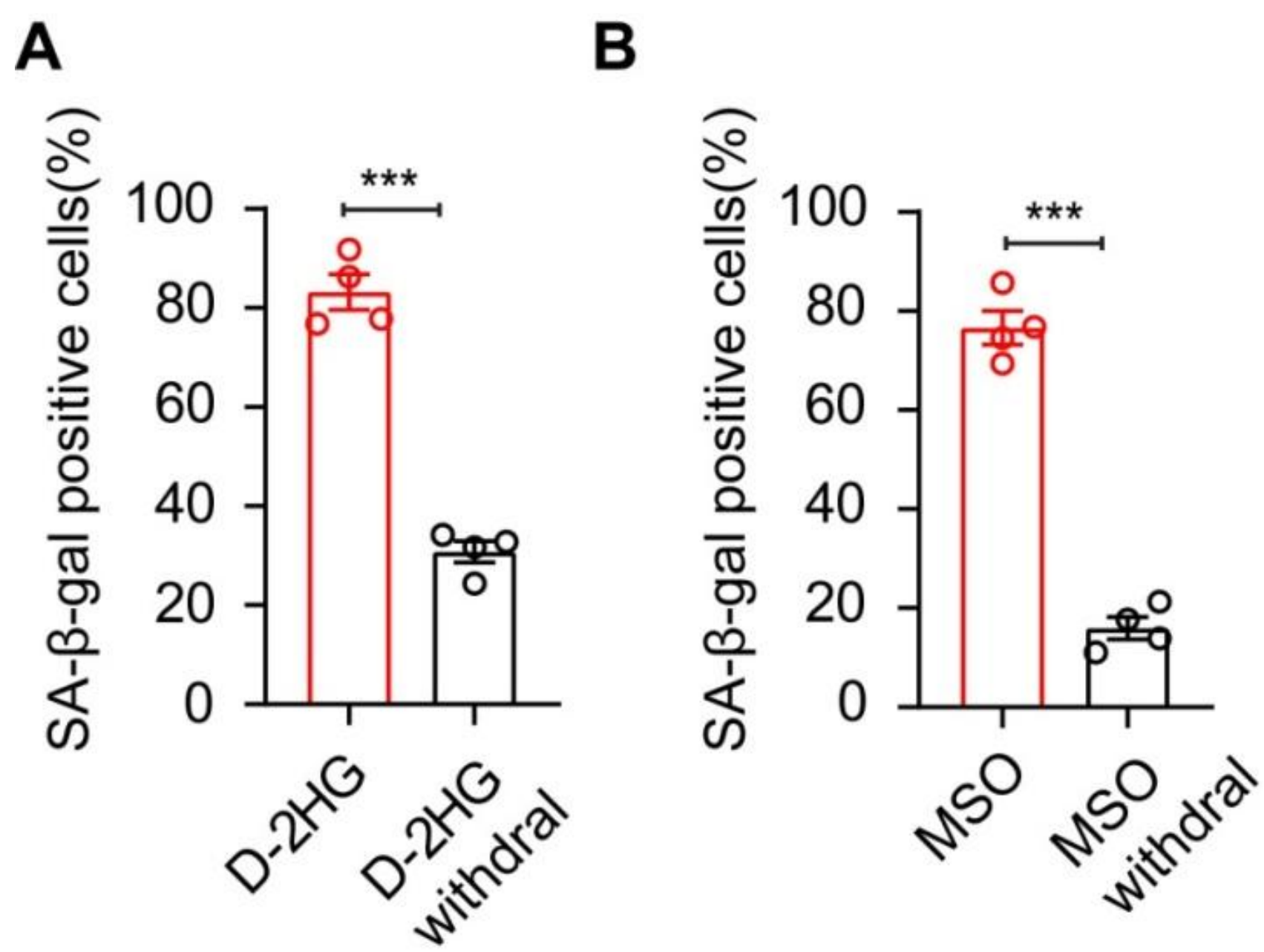

Supplementary Figure 4. Cellular senescence induced by D-2HG or MSO is reversible. (**A**) U87 glioma cells were pretreated with 20 mM D-2HG for 72 hours. The percentage of SA-β-gal-positive cells was subsequently quantified either after continuous exposure to D-2HG or following D-2HG withdrawal for an additional 72 hours. (**B**) U87 glioma cells were pretreated with 5 mM MSO for 72 hours. The percentage of SA-β-gal-positive cells was subsequently quantified either after continuous exposure to MSO or following MSO withdrawal for an additional 72 hours. The data are representative of three independent experiments with n = 4 per group and were normalized to the total cell number. All the data are presented as means ± SEMs. Statistical significance was determined using unpaired Student's t tests (*p < 0.05, **p < 0.01, ***p < 0.001).

© 2025. Zhao Y et al. Published online at http://www.aginganddisease.org/EN/10.14336/AD.2025.0554
